# Physicochemical properties of vitreous and floury endosperm flours in maize

**DOI:** 10.1002/fsn3.1114

**Published:** 2019-07-04

**Authors:** Haiyan Zhang, Guanghai Xu

**Affiliations:** ^1^ College of Agronomy Qingdao Agricultural University Qingdao China

**Keywords:** flour, floury endosperm, maize, physicochemical properties, vitreous endosperm

## Abstract

Three maize cultivars with different endosperm types (flint, semiflint, and dent maize cultivars) were studied to characterize vitreous endosperm flour properties compared with those of floury endosperm flour from the same maize kernels. Vitreous endosperm flour had higher amylose and protein contents, and lower starch content, higher percentage of large starch granule, bigger mean diameter of starch granule, higher iodine capacity, higher trough viscosity and final viscosity and setback, lower swelling power, lower peak viscosity and breakdown, and higher peak time and pasting temperature than the counterpart floury endosperm flour. X‐ray diffraction analysis indicated typical A‐pattern for starches of vitreous and floury endosperm flours. Floury endosperm flour showed the presence of greater crystallinity and higher enthalpy change (*∆Hgel*) than vitreous endosperm flour for three cultivars. Retrogradation enthalpy (*∆Hret*) and retrogradation percentage (*R*) of vitreous and floury endosperm flours ranged from 6.23 to 7.92 J/g and 52.72% to 73.62%, and from 5.46 to 6.45 J/g and 45.70% to 56.58%, respectively. In conclusion, vitreous and floury endosperm flours had significantly different physicochemical properties. Results of this study provide a foundation for better and valid utilization of different endosperm section during grain processing.

## 1 INTRODUCTION

Maize (*Zea mays* L.), one of the worldwide crops, has been applied in many areas such as feeding, food, and chemicals (Gayral et al., 2016). The maize kernel is made up of three main sections: pericarp, embryo, and endosperm. Endosperm is the major component of maize kernel, representing 80%–85% of the dry weight. Endosperm type decides the texture of the kernel, which is a significant feature for maize grain quality because it affects the shipping and handling characteristics of the grain, the susceptibility to insects, the grits yield from dry milling, the energy costs during wet milling, and the cooking properties of the flour (Zhang, Gao, & Dong, 2011). Vitreousness, an important factor to assess grain texture, is usually determined by the ratio of vitreous and floury endosperm based on the endosperm cut surface appearance (Baasandorj, Ohm, & Simsek, 2016). The vitreous region, usually in the periphery of the endosperm, is glasslike and translucent, whereas floury endosperm, usually in the center of the endosperm, is white, mealy, and nontranslucent (Baasandorj et al., 2016).

Vitreousness reflects the compactness of the starch–protein matrix (Gayral et al., 2016). Vitreous endosperm with the compacted and well‐developed protein matrix (Zhang et al., 2011) produces hard kernels, and this type of maize kernel is often used for nixtamalized and extruded products (Juárez‐García, Agama‐Acevedo, Gómez‐Montiel, Pando‐Robles, & Bello‐Pérez, 2013). On the contrary, floury endosperm with discontinuous protein matrix (Gayral et al., 2016) causes soft kernels, and this type of maize kernel is usually applied to produce flour (Juárez‐García et al., 2013) and feeding (Rossi et al., 2016) since the kernels break apart easily. In order to well direct the use for maize endosperm, there is a need to characterize their properties and differences between vitreous and floury endosperms.

The hardness distinction between the two types of endosperm may impact on the nutritive value of maize (Tamagno et al., 2016), and the flour behavior and functionality (Narváez‐González, Figueroa‐Cárdenas, Taba, & Sánchez, 2006). Gayral et al. (2015, 2016) found that there were higher amylose and protein contents within starches of vitreous than those of floury endosperms. The pasting and thermal properties of flours provide valuable information about their functionality. Peak viscosity shows the point at which most starch granules attain a maximum swelling, after which a collapse happens. It is therefore an indicator of water retention ability (Beta, Corke, Taylor, & Rooney, 2001). Gelatinization temperature indicates the dissociation of the double helix of amylopectin and the fusion of starch crystals (Sasaki & Matsuki, 1998). The previous study found that highly compact kernels developed low peak and final viscosities, small and polygonal starch granules, and requirement of more time and higher temperature for gelatinization (Narváez‐González et al., 2006).

According to the variations of the vitreous and floury portions in maize kernel, hybrids are classified into flint maize, semiflint maize, and dent maize. However, the physicochemical properties of vitreous and floury endosperm flours from the same kernels for these three types of maize have not been analyzed. The aim of this work was to investigate the physicochemical properties of vitreous and floury endosperm flours with different endosperm types of maize.

## 2 MATERIALS AND METHODS

### 2.1 Plant material

Flint maize (Xianyu 335, vitreousness: 60.0%), semiflint maize (Qingnong 105, vitreousness: 43.8%), and dent maize (Longping 206, vitreousness: 20.0%) were planted at 67,500 plants/ha on 20 June 2017 in Qingdao Agricultural University, Shandong, China. The experiment was a randomized complete block design with three replications. Every plot comprised of ten rows, 0.60 m spacing between rows and 8.0 m long. Maize kernels at the middle position on an ear from the same hybrid plants were collected.

### 2.2 Vitreousness determination and flour preparation of vitreous and floury endosperm

Vitreousness was determined according to the method of Zhang et al. (2011). To prepare vitreous and floury endosperm flours, after the whole kernels were soaked for 24 hr with deionized water, the pericarp, aleurone, embryo, and tip cap were removed manually using a blade and tweezers to obtain endosperm which would experience a longitudinal cut. The vitreous and floury fractions were isolated using the hand dissection and dried at 40°C. After drying, vitreous endosperm and floury endosperm were ground to homogeneous flour using a grinder and passed through 150‐μm sieve for physicochemical analysis.

### 2.3 Amylose, starch, and protein content

Amylose content was measured using the colorimetric method described by Knutson (1986). Starch content was evaluated using the anthrone–sulfuric acid method (Hansen & Møller, 1975). Nitrogen content was determined using the Kjeldahl method (Zhang et al., 2011), and protein content was estimated using the nitrogen‐to‐protein conversion factor of 6.25.

### 2.4 Starch granule size distribution

After the flour samples were dyed with I_2_‐KI, starch granule diameter was measured by 15× eyepiece and 40× objective with Olympus microscope. More than 100 starch granules were determined for every field. Every sample was made for three fields. Starch granule mean diameter and size distribution were calculated.

### 2.5 Iodine staining

The maximum absorption wavelength (λ_max_) and blue value of flours were determined as described by Fiedorowicz and Rebilas (2002). 40 mg flour was added to 10 ml of DMSO containing 10% of 6 M urea. A 1.0 ml aliquot was placed in a 100‐ml volumetric flask, to which 95 ml of deionized water and 2 ml of an aqueous I_2_‐KI solution were added. The mixture was made up to 100 ml with deionized water and mixed immediately. Blank solution was prepared identically without flour. Spectra in the range of 500–700 nm were measured using a UV–visible spectrophotometer. The blue value was defined as the absorbance at 635 nm, and the λ_max_ was the peak absorbance value over the range of wavelengths examined. The iodine binding capacity was the ratio of absorbance at 635 nm to that at 520 nm.

### 2.6 Swelling power

Swelling power was determined as described by McCormick, Panozzo, and Hong (1991). 0.25 g flour was weighed into a plastic centrifuge tube, and 5 ml distilled water was added. The tube contents were mixed and heated in a shaking water bath at 70°C for 4 min. The contents were mixed again, and were transferred to a second shaking water bath at 70°C for 6 min and a third boiling water bath for 10 min. The tube was cooled to room temperature in cold water and centrifuged at 4,000×*g* for 20 min. The supernatant was discarded, and the tube was weighed. The sediment weight was calculated, and swelling power was determined as sediment weight divided by dry sample weight.

### 2.7 Pasting properties

The pasting properties of the flours were evaluated using a rapid viscosity analyzer (RVA‐TecMaster; Newport Scientific) as described by Lu and Lu (2012). 3.5 g flour was dispersed in 25 g distilled water. The obtained suspension was equilibrated at 50°C for 1 min, heated to 95°C at 12°C/min, maintained at 95°C for 2.5 min, cooled to 50°C at 12°C/min, and then maintained at 50°C for 1 min. The paddle speed was set at 960 rpm for the first 10 s and then decreased to 160 rpm for the rest of the analysis.

### 2.8 X‐ray diffraction pattern

The X‐ray diffraction (XRD) patterns of the samples were achieved using an X‐ray diffractometer (D8 Advance, Bruker AXS) as described by Lu and Lu (2012). The diffractometer was operated at 40 mA and 40 kV. The scanning region of the diffraction angle (2θ) ranged from 5° to 45° at a step size of 0.02° with a counting time of 0.1 s. Relative crystallinity (%) was calculated as the ratio of the crystallinity area to the total diffraction area following the method described by Wei et al. (2010).

### 2.9 Thermal properties

The thermal characteristics of the flours were determined by differential scanning calorimetry (DSC1; Mettler‐Toledo) as described by Lu and Lu (2012). 5 mg sample was weighed into an aluminum pan. Distilled water was added to obtain a flour–water suspension containing 66.7% water. The pan was sealed tightly and allowed to stand for 12 hr at 4°C before further analysis. Empty aluminum pan was used as reference. Sample pan was heated at a rate of 10°C/min from 20 to 100°C. Onset temperature (*To*), peak temperature (*Tp*), conclusion temperature (*Tc*), and enthalpy change (Δ*H_gel_*) of flour samples were calculated automatically. After thermal analysis, the samples were kept at 4°C for 7 days for retrogradation analysis. The sample pan containing the flour was reheated at the rate of 10°C/min from 20 to 100°C to measure retrogradation. The retrogradation enthalpy (Δ*Hret*) was evaluated automatically, and retrogradation percentage (%*R*) was calculated as %*R* = 100 × Δ*Hret*/Δ*Hgel*.

### 2.10 Statistical analysis

The data were subjected to analysis of variance by LSD test at the 5% probability level using the DPS software.

## 3 RESULTS AND DISCUSSION

### 3.1 Amylose, starch, and protein content

Amylose level was significantly higher in samples from vitreous endosperm than those from corresponding floury endosperm (Table 1), which is also confirmed by Gayral et al. (2015). For the same endosperm type, amylose content of three cultivars appeared as Xianyu 335 > Qingnong 105 > Longping 206 (Table 1). This result was consistent with the higher amylose contents of a quality protein maize (QPM) population with increased vitreousness (Dombrink‐Kurtzman & Knutson, 1997). It was found that the increase in amylose content was in accordance with the increase in the activity of granule‐bound starch synthase type I (GBSSI), which was the primary enzyme for amylose biosynthesis in the endosperm (Vrinten & Nakamura, 2000). The percentage of amylose in starch was always greater in samples from hard endosperm than in the samples from corresponding soft endosperm (Dombrink‐Kurtzman & Knutson, 1997), consistent with our finding. Different isoforms of GBSS were found in the endosperm (GBSSI) and pericarp (GBSSII) of wheat, explicating the different amylose content in these tissues (Vrinten & Nakamura, 2000). Perhaps different isoforms of GBSS were present in different sections of maize endosperm that caused the differences in amylose content between vitreous endosperm and floury endosperm.

**Table 1:** Amylose, starch, and protein content of vitreous and floury endosperm flours from different maize cultivars

Cultivar	Endosperm type	Amylose content (%)	Starch content (%)	Protein content (%)
Xianyu 335	Vitreous endosperm	25.41 ± 0.4^a^	72.10 ± 0.5^d^	10.37 ± 0.2^a^
	Floury endosperm	20.96 ± 0.5^c^	74.51 ± 0.3^b^	9.18 ± 0.1^c^
Qingnong 105	Vitreous endosperm	23.44 ± 0.3^b^	72.06 ± 0.1^d^	10.57 ± 0.4^a^
	Floury endosperm	19.92 ± 0.7^d^	75.36 ± 0.4^a^	8.96 ± 0.2^cd^
Longping 206	Vitreous endosperm	21.79 ± 0.3^c^	73.21 ± 0.3^c^	9.93 ± 0.1^b^
	Floury endosperm	18.16 ± 0.4^e^	75.89 ± 0.4^a^	8.80 ± 0.1^d^

Note

Data are expressed as mean ± *SD*. Values in the same column followed by different letters are significantly different (*p* < 0.05).

As previously shown in opaque and inbred maize line (Dombrink‐Kurtzman & Bietz, 1993; Landry, Delhaye, & Damerval, 2004), higher starch content was found in the floury endosperm (Table 1). In this study, higher protein content was observed in the vitreous endosperm (Table 1), which was consistent with that hard endosperm contains more protein bodies (Pereira et al., 2008) which were primarily due to α‐zeins (Dombrink‐Kurtzman & Bietz, 1993; Gayral et al., 2016).

### 3.2 Starch granule size distribution

No significant differences in starch granule mean diameter can be observed among three cultivars (Table 2). However, significant differences were observed between the vitreous and floury region of the same cultivar (Table 2). The vitreous region had much larger starch granule and bigger mean diameter of starch granule than floury region, which was consistent with the larger particles in the flint line (Gayral et al., 2016), but was inconsistent with the large starch granules in low compact kernels (Narváez‐González et al., 2006). In maize kernel, floury endosperm is farther than vitreous endosperm from aleurone, which causes later amyloplast development in floury endosperm cell than vitreous endosperm cell. It was supposed that amyloplast development time affected starch granule size. The earlier amyloplast development led to larger starch granule diameter (Yi & Zhang, 2014).

**Table 2:** Starch granule size distribution of vitreous and floury endosperm flours from different maize cultivars

Cultivar	Endosperm type	The distribution of different starch granule (%)								Mean diameter (µm)
		≤6 µm	(6, 9) µm	(9, 12) µm	(12, 15) µm	(15, 18) µm	(18, 21) µm	(21, 24) µm	>24 µm	
Xianyu 335	Vitreous endosperm	0	0.6	3.4	24	45.6	20.2	2.8	3.4	16.50 ± 0.1^a^
	Floury endosperm	0.8	11.2	13.6	28	35	8.6	1.8	1	13.85 ± 0.2^b^
Qingnong 105	Vitreous endosperm	0	0.6	3.8	16	37.2	35.4	5.2	1.8	16.90 ± 0.1^a^
	Floury endosperm	1.4	17.8	19.8	22	23.8	13.8	0.2	1.2	12.92 ± 0.2^b^
Longping 206	Vitreous endosperm	0	0.8	8.2	15	36.8	31	5	3	16.73 ± 0.2^a^
	Floury endosperm	1.6	13.8	22.6	20.8	32.2	7.6	1.4	0	13.20 ± 0.2^b^

Note

Data are expressed as mean ± *SD*. Values in the same column followed by different superscript letters are significantly different (*p* < 0.05).

### 3.3 Iodine staining and swelling power

Blue value of vitreous endosperm was significantly higher than that of floury endosperm for the tested cultivars except for Longping 206 (Table 3). The iodine binding capacity of vitreous endosperm was significantly higher than that of floury endosperm (Table 3). The iodine binding capacity can be used to estimate the amylose content or the ratio of long to short chains in amylopectin (Fiedorowicz & Rebilas, 2002). In this study, high iodine binding capacity values of vitreous endosperm were consistent with not only its high amylose content (Table 1) but also larger amount of long chains in the amylopectin in vitreous endosperm (Juárez‐García et al., 2013). The *λ*_max_ values ranged from 596.2 to 601.4 nm, which was not significantly different between vitreous endosperm and floury endosperm for three cultivars (Table 3).

**Table 3:** Iodine staining and swelling power of vitreous and floury endosperm flours from different maize cultivars

Cultivar	Endosperm type	Blue value	*λ*_max_ (nm)	Iodine binding capacity	Swelling power (g/g)
Xianyu 335	Vitreous endosperm	0.590 ± 0.012^a^	600.9 ± 1.9^ab^	1.553 ± 0.03^b^	10.25 ± 0.3^d^
	Floury endosperm	0.548 ± 0.001^c^	601.4 ± 0.0^a^	1.450 ± 0.01^c^	14.00 ± 0.4^b^
Qingnong 105	Vitreous endosperm	0.568 ± 0.002^b^	598.7 ± 1.5^ab^	1.760 ± 0.05^a^	10.66 ± 0.4^d^
	Floury endosperm	0.549 ± 0.001^c^	596.2 ± 0.0^b^	1.353 ± 0.01^d^	14.78 ± 0.5^a^
Longping 206	Vitreous endosperm	0.563 ± 0.003^b^	597.4 ± 5.5^ab^	1.423 ± 0.06^c^	11.60 ± 0.2^c^
	Floury endosperm	0.565 ± 0.004^b^	601.1 ± 2.9^a^	1.283 ± 0.03^e^	13.55 ± 0.3^b^

Note

Data are expressed as mean ± *SD*. Values in the same column followed by different superscript letters are significantly different (*p < *0.05).

Swelling power of flour from different maize cultivars varied between 10.25 and 14.78 g/g. Floury endosperm flour showed higher swelling power (13.55–14.78 g/g) than vitreous endosperm flour (10.25–11.60 g/g; Table 3). In cereal starches, swelling of starch granules is an attribute of amylopectin, as amylose actively restrains swelling (Tester & Morrison, 1990). Villanueva, Lamo, Harasym, and Ronda (2018) observed that proteins retained water from the starch granules and consequently reduced the swelling. Li, Wang, Chen, Yu, and Feng (2018) found that decreased protein content significantly weakened starch–protein network interactions, thereby resulting in increased swelling power. Thus, the amount of swelling was inversely related to the amylose and protein contents. In this study, both higher amylose and protein contents (Table 1) contributed to lower swelling power of vitreous endosperm.

### 3.4 Pasting properties

Results of pasting properties of three cultivars are summarized in Table 4. Floury endosperm flour had higher peak viscosity (PV) and breakdown (BD), and lower trough viscosity (TV), final viscosity (FV), and setback (SB) than vitreous endosperm flour. Pasting properties were regulated by the amylose content, starch granule size, and chain‐length distribution of amylopectin (Jane et al., 1999). PV and BD were negatively correlated with starch granule size and the ratio of long chain, and positively correlated with starch content (Lu et al., 2013). The values of PV and BD were also either increased or decreased, depending on protein type and its concentration (Li et al., 2018; Noisuwan, Bronlund, Wilkinson, & Hemar, 2008). It was hypothesized that certain proteins formed a matrix and then restrain swelling, and consequently, lower viscosity was observed with higher protein content (Li et al., 2018; Mansilla, Nazar, & Pérez, 2017). Furthermore, as the dilution factor, proteins reduced the starch water absorption with the ensuing decrease in viscosity (Baxter, Blanchard, & Zhao, 2014; Mansilla et al., 2017). Therefore, the lower PV and BD of vitreous endosperm flour should relate to the bigger granules (Table 2), higher ratio of long chain in the starch (Table 3), and higher protein content and lower starch content (Table 1). In this study, the higher FV and SB of vitreous endosperm flour were contrary to the results that very compact kernels developed low FV and SB (Narváez‐González et al., 2006), possibly because the microstructure is different during and after pasting compared to before and the tested sample origin is also different. But this was consistent with the reports that FV increase was because of the aggregation of the amylose molecules (Olu‐Owolabi, Afolabi, & Adebowale, 2011). Peak time (PT) and pasting temperature (P_temp_) of vitreous and floury endosperm flour ranged from 4.67 to 4.73 min and 71.00 to 72.55°C, and from 4.00 to 4.47 min and 69.35 to 70.25°C, respectively (Table 4). Floury endosperm flour showed a lower PT and P_temp_ than vitreous endosperm flour, which was similar to highly compact kernels with a high percentage of hard endosperm requiring more time and higher temperature for gelatinization (Mansilla et al., 2017; Narváez‐González et al., 2006). In vitreous endosperm, protein matrix clung to the surface of starch granules (Zhang et al., 2011). This indicated that the protein matrix of vitreous endosperm means a physical barrier to water migration into starch granules, delaying the pasting and gelatinization process.

**Table 4:** Pasting properties of vitreous and floury endosperm flours from different maize cultivars

Cultivar	Endosperm type	PV (RVU)	TV (RVU)	BD (RVU)	FV (RVU)	SB (RVU)	PT (min)	*P*_temp_ (°C)
Xianyu 335	Vitreous endosperm	210.7 ± 12.4^d^	97.8 ± 3.3^bc^	112.9 ± 9.1^c^	202.7 ± 5.1^bc^	104.9 ± 1.8^a^	4.73 ± 0.04^a^	72.55 ± 0.4^a^
	Floury endosperm	248.4 ± 6.1^c^	63.2 ± 1.1^d^	185.3 ± 7.2^a^	121.2 ± 1.4^e^	58.0 ± 2.5^d^	4.00 ± 0.00^d^	70.25 ± 0.1^cd^
Qingnong 105	Vitreous endosperm	139.6 ± 3.8^e^	150.3 ± 1.4^a^	76.7 ± 2.5^d^	259.6 ± 4.2^a^	109.3 ± 2.8^a^	4.67 ± 0.04^b^	71.00 ± 0.2^bc^
	Floury endosperm	310.5 ± 6.7^a^	62.9 ± 1.9^d^	160.3 ± 8.7^b^	132.8 ± 6.4^d^	69.9 ± 8.3^c^	4.47 ± 0.06^c^	69.45 ± 0.3^d^
Longping 206	Vitreous endosperm	192.3 ± 3.3^d^	97.6 ± 3.5^b^	94.7 ± 0.2^d^	210.0 ± 6.0^b^	112.4 ± 2.4^a^	4.67 ± 0.00^b^	71.75 ± 0.1^ab^
	Floury endosperm	288.8 ± 6.1^b^	94.6 ± 3.1^c^	194.3 ± 3.1^a^	191.1 ± 1.8^c^	96.5 ± 1.3^b^	4.00 ± 0.00^d^	69.35 ± 0.1^d^

Note

Data are expressed as mean ± *SD*. Values in the same column followed by different superscript letters are significantly different (*p* < 0.05).

Abbreviations: BD, breakdown; FV, final viscosity; *P*_temp_, pasting temperature; PT, peak time; PV, peak viscosity; SB, setback; TV, trough viscosity.

### 3.5 X‐ray diffraction

**Figure 1:**
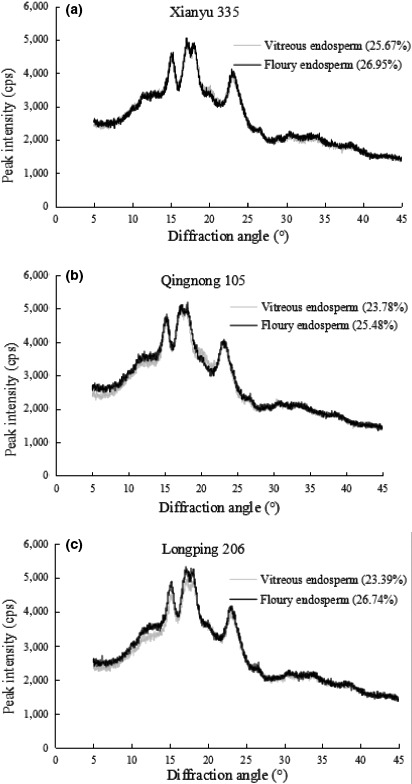
X‐ray diffraction pattern and crystallinity of vitreous and floury endosperm flours from different maize cultivars

X‐ray diffraction patterns of the samples are illustrated in Figure 1. The results indicated that the starches of vitreous and floury endosperm flours were typical A‐pattern which means a single peak at 2θ = 15° and 23°, and dual peaks at 2θ = 17–18°. Besides 2θ = 20°, peak intensities at 15°, 17°, 18°, and 23° were significantly higher floury endosperm flour than vitreous endosperm flour for three cultivars (Figure 1). The additional peak at 2θ = 20° has been attributed to amylose–lipid complexes according to the previous study (Zobel, 1988). Furthermore, vitreous endosperm had higher amylose content (Table 1). Therefore, the presence of larger number of amylose–lipid complexes in vitreous endosperm flour caused its higher peak intensity at 2θ = 20°.

The differences in crystallinities between endosperm types were the same as the peak intensities except for 2θ = 20°, because those parameters were positively correlated between each other according to the previous results (Lu et al., 2013). Crystallinity was affected by amylose content (Cheetham & Tao, 1998; Singh, Inouchi, & Nishinari, 2006). Singh et al. (2006) found that waxy maize starch without any amylose showed maximum crystallinity, whereas sugary maize starch with the highest amylose showed minimum crystallinity. In this study, the lower crystallinity of starch in vitreous endosperm flour agreed with the higher amylose content of vitreous endosperm (Table 1).

### 3.6 Thermal properties

In this study, the transition temperatures of the endotherm (onset temperature, *To*; peak temperature, *Tp*; conclusion temperature, *Tc*) and enthalpy change (*∆Hgel*) of vitreous endosperm flour from three maize cultivars ranged between 63.70 and 64.32°C, 69.16 and 69.70°C, and 75.03 and 76.61°C, and between 8.57 and 12.58 J/g, respectively, while floury endosperm flour had a range of 64.39–65.64, 69.72–69.87, and 75.64–77.02°C, and 9.65–12.90 J/g, respectively (Table 5). Vitreous endosperm flour showed lower transition temperatures and *∆Hgel*, which may be due to the presence of lower crystallinity indicating the presence of more amorphous region (Barichello, Yada, Coffin, & Stanley, 1990; Singh et al., 2006). According to the former study, these differences in calorimetric results between vitreous and floury endosperm flours may be also due to water competition phenomena between starch and other components and denaturation of proteins (Bravo‐Núñez & Gómez, 2019; Torres, Moreira, Chenlo, & Morel, 2013). Retrogradation enthalpy (*∆Hret*) of vitreous and floury endosperm flours ranged between 6.23 and 7.92 J/g and between 5.46 and 6.45 J/g, respectively. Their retrogradation percentage (*R*) ranged between 54.06%–73.62% and 44.54%–56.59%, respectively. And the higher *∆Hret* and *R* were recorded in the vitreous endosperm flour, and the lower in floury endosperm flour (Table 5). These results may be related to the compactness of starch granules in the vitreous endosperm, which on the one hand decreased the mobility of the linear chains of starch and on the other hand increased flour retrogradation (Yuan, Thompson, & Boyer, 1993).

**Table 5:** Thermal properties of vitreous and floury endosperm flours from different maize cultivars

Cultivar	Endosperm type	*∆Hgel* (J/g)	*To* (°C)	*Tp* (°C)	*Tc* (°C)	*∆Hret* (J/g)	*R* (%)
Xianyu 335	Vitreous endosperm	12.58 ± 0.41^a^	63.70 ± 0.24^c^	69.70 ± 0.14^a^	76.61 ± 0.14^b^	6.80 ± 0.25^b^	54.06 ± 2.75^bc^
	Floury endosperm	12.90 ± 0.50^a^	64.39 ± 0.64^bc^	69.72 ± 0.07^a^	77.02 ± 0.06^a^	5.75 ± 0.37^cd^	44.54 ± 5.18^d^
Qingnong 105	Vitreous endosperm	8.57 ± 0.37^d^	64.32 ± 0.38^bc^	69.33 ± 0.06^b^	75.03 ± 0.10^e^	6.23 ± 0.13^bc^	72.82 ± 1.21^a^
	Floury endosperm	9.65 ± 0.34^c^	64.89 ± 0.60^ab^	69.86 ± 0.13^a^	75.64 ± 0.08^c^	5.46 ± 0.30^d^	56.59 ± 4.44^b^
Longping 206	Vitreous endosperm	10.76 ± 0.50^b^	63.88 ± 0.93^bc^	69.16 ± 0.13^b^	75.38 ± 0.12^d^	7.92 ± 0.34^a^	73.62 ± 1.29^a^
	Floury endosperm	12.76 ± 0.85^a^	65.64 ± 0.85^a^	69.87 ± 0.07^a^	75.76 ± 0.07^c^	6.45 ± 0.47^b^	50.72 ± 1.40^c^

Note

Data are expressed as mean ± *SD*. Values in the same column followed by different superscript letters are significantly different (*p* < 0.05).

Abbreviations:* ∆Hgel*: gelatinization enthalpy; *∆Hret*: retrogradation enthalpy; *R*: retrogradation percentage; *To*: onset temperature; *Tp*: peak gelatinization temperature; *Tc*: conclusion temperature.

## 4 CONCLUSION

The findings in the current study revealed that the physicochemical properties of floury endosperm flour samples of three maize cultivars differed from their counterpart vitreous endosperm flour samples. Due to poor agronomic traits and nutritional quality, floury endosperm is usually undesirable. In this study, the higher swelling power, higher pasting peak viscosity, and lower setback and lower pasting temperature and less peak time, higher crystallinity, and lower retrogradation level compared to vitreous endosperm flour make floury endosperm flour a potentially new functional ingredient. Therefore, either floury endosperm or vitreous endosperm flour has great potential to play the special role in the food industry.

## CONFLICT OF INTEREST

The authors declare that they do not have any conflict of interest.

## ETHICAL APPROVAL

This study does not involve any human or animal testing.
